# Classification and Differential Diagnosis of Diabetic Nephropathy

**DOI:** 10.1155/2017/8637138

**Published:** 2017-02-20

**Authors:** Chenyang Qi, Xing Mao, Zhigang Zhang, Huijuan Wu

**Affiliations:** ^1^Department of Pathology, Shanghai Medical College, Fudan University, Shanghai, China; ^2^Shanghai Institute for Kidneys and Dialysis, Shanghai, China

## Abstract

Diabetic nephropathy (DN) is a major cause of end-stage renal disease throughout the world in both developed and developing countries. This review briefly introduces the characteristic pathological changes of DN and Tervaert pathological classification, which divides DN into four classifications according to glomerular lesions, along with a separate scoring system for tubular, interstitial, and vascular lesions. Given the heterogeneity of the renal lesions and the complex mechanism underlying diabetic nephropathy, Tervaert classification has both significance and controversies in the guidance of diagnosis and prognosis. Applications and evaluations using Tervaert classification and indications for renal biopsy are summarized in this review according to recent studies. Meanwhile, differential diagnosis with another nodular glomerulopathy and the situation that a typical DN superimposed with a nondiabetic renal disease (NDRD) are discussed and concluded in this review.

## 1. Introduction

Diabetic nephropathy (DN) caused by diabetes mellitus is one of the major causes of end-stage renal failure worldwide [1]. Clinically, microalbuminuria is an important index to assess the progression of DN [2]. However, it is not accurate to evaluate the severity or prognosis simply based on the degree of proteinuria. It is now well recognized that not all diabetic patients who develop renal function failure have massive albuminuria [3]. Therefore, nephrologists and endocrinologists should be aware of the significance of pathological changes of DN in their clinical practice. Specifically, nondiabetic renal disease (NDRD), which might commonly be superimposed with diabetic renal lesions in some patients with type 2 diabetes, could only be confirmed and excluded by biopsy [4].

## 2. Pathological Changes of DN

The most significant and consistent pathological changes identified in renal biopsies of clinical DN patients are the glomerular lesions [5] which are, especially, diffuse and nodular mesangial expansion and Glomerular Basement Membrane (GBM) thickening [6]. Diffuse mesangial expansion, which develops at as early as 5th year since the onset of diabetes, is the earliest observable change by light microscopy [7]. The mesangial fractional volume [Vv(Mes/glom)] is correlated with albumin excretion rate (AER) and Glomerular Filtration Rate (GFR) in both type 1 [8] and type 2 diabetes [9]. As the disease advances, diffuse mesangial expansion progressively develops into nodular accumulations of mesangial matrix in the late stage of the DN. These nodular lesions, also known as Kimmelstiel-Wilson nodules, can be observed in about 25% of patients with advanced DN [10]. Nodular lesions and diffuse lesions are two stages of DN. Compared to the patients with diffuse mesangial expansion, those patients with nodular diabetic glomerulosclerosis present more severe renal damage, longer diabetic durations, and poorer renal prognosis [11].

GBM thickening can be observed within 2–8 years after the onset of diabetes. It is an early lesion which could be detected and measured by electron microscopy (EM) [12]. GBM width tends to increase linearly according to diabetes duration in type 1 diabetes [13]. Vv(Mes/glom) and GBM width together explain 59% of the AER variability in a group of 125 patients of type 1 diabetes [8].

Although diabetic glomerular lesions have been the focus of the investigation on DN, the extraglomerular lesions are also involved in the progression of the disease. Tubulointerstitial lesions, including tubular atrophy, interstitial inflammation, and tubulointerstitial fibrosis, are closely related to renal function loss in the progression towards ESRD in patients with preexisting renal insufficiency [14]. Since DN is a kind of diabetic microangiopathy, hyalinosis occurs in both afferent and efferent arterioles. The hyalinosis of the efferent arteriole is a typical lesion by which diabetic nephropathy could be differentiated from hypertensive nephropathy [15].

There is increasing recognition of lesions like glomerular endothelial injury [5], podocyte impairment [16], and glomerulotubular junctions abnormalities in DN [17]. Given that the detection methods of these lesions are difficult to generalize in clinical practice, for now their value in diagnosis and classification is not as important as glomerular, tubulointerstitial, and vascular lesions.

## 3. Tervaert Classification of Diabetic Nephropathy

Journal of the American Society of Nephrology published pathological classification of DN by Tervaert et al. in 2010 (Tables 1 and 2) [10].

## 4. The Application and Evaluation of Tervaert Classification of DN

### 4.1. Contribution to the Early Diagnosis of Diabetic Nephropathy and Guiding Significance for Renal Prognosis in Patients with Diabetes

The classification outlined in Tervaert et al. is based on glomerular lesions, which best reflect the course of progressive DN. This is an important first step to set up an evaluable scheme of clinical value. Patients with typical DN show a longer impairment duration, worse metabolic control, and higher prevalence of diabetic retinopathy [18]. Diabetic patients with microalbuminuria without glomerulopathy are more likely to either regress to normoalbuminuria or remain with microalbuminuria with a slower rate of decline in renal function [19].

Using Tervaert pathological classification, Zhu et al. rediagnosed 37 cases of renal biopsies obtained from patients with type 2 diabetes manifesting microalbuminuria or clinical albuminuria and found that 6 out of 11 patients who were previously diagnosed with nondiabetic nephropathy actually belonged to class I~class II DN [20]. To explore the significance of this classification on prognosis, one group analyzed the relationship between structural changes and clinical features in 50 patients with type 2 diabetic and found that, as the glomerular lesions advanced from classes I to IV, the average GFR and 5-year renal survival decreased (100% of renal survived in classes I and IIa patients and 75% and 66.7% in classes IIb and III patients, respectively, whereas only 38.1% in class IV patients) [21]. Furthermore, another large-scale follow-up study of 396 patients with type 2 diabetes also revealed that the severity of glomerular and interstitial lesions had a significant impact on renal prognosis and could be used as an independent risk factor for renal outcomes [22]. Studies mentioned above all employed Tervaert pathological classification of DN, which suggested that this method can help to increase the accuracy of diagnosis, the possibility of an early diagnosis, and treatment of DN, as well as the prediction of renal prognosis.

As interstitial lesions also contribute to the impairment in renal function and may be an independent factor involved in the progression of DN, the classification scheme has put the extraglomerular lesions into consideration and introduces a separate scoring system for interstitial and vascular lesions.

### 4.2. Requirements to Involve Tubular, Interstitial, and Vascular Lesions into the Classification

Although the severity of tubular, interstitial, and vascular lesions has been taken into consideration by Tervaert classification, it is still not enough to meet practical requirements. Before Tervaert classification, Fioretto classification has been used for a long time, which included tubular, interstitial, and vascular lesions and divided DN into 3 categories according to the pathological changes under light microscope: C1, normal/near normal; C2, typical diabetic nephropathy with predominantly glomerular changes; and C3, atypical patterns of injury, associated with disproportionately damage including tubulointerstitial or arteriolar hyalinosis and with absent or only mild diabetic glomerular changes [23]. By using Fioretto classification, 31 patients with type 2 diabetes accompanied with normo-, micro-, or macroalbuminuria were investigated, and GFR lower than 60 mL/min/1.73 m^2^ was considered as the decline of renal function [24]. Majority of patients (22 of 23) with micro- or macroalbuminuria were diagnosed as typical glomerular changes (C2) of Fioretto classification, and only one patient was diagnosed as atypical pattern of renal damage (C3). Notably, among patients with normoalbuminuria, 3 of 8 were diagnosed as C3 according to the tubular, interstitial, and vascular lesions [24]. When compared to Fioretto classification, Tervaert classification was only based on glomerular lesions, which is not sufficient for clinical application, which failed to classify tubular, interstitial, and vascular lesions belonging to Fioretto C3, due to the heterogeneity of the renal lesions and the complicated mechanism underlying diabetic nephropathy.

### 4.3. The Complexity and Heterogeneity of Type 2 Diabetes Should be Involved in Classification

Renal lesions in type 2 diabetes are much more complex than those in type 1 diabetes. There are more challenges to reach a correlation or predictive accuracy in renal function with glomerular structural variables in type 2 diabetes than in type 1 diabetes [19]. Tervaert et al. suggested that their classification can be used for both type 1 and type 2 diabetic patients, because of the substantial overlaps between these two types in histologic changes and clinical complications [10]. However, failure to distinguish the patients with type 1 diabetes or with type 2 diabetes in Tervaert classification might limit its significance on clinical practice [25]. There are two points worth taking into account for type 2 diabetes.

The first point is the high prevalence of nondiabetic superimposed renal lesions in type 2 diabetes. In many clinical cases, renal biopsies are usually performed in patients with an atypical manifestation of DN. There is no wonder that nondiabetic renal lesions in proteinuric type 2 diabetic patients have a prevalence as high as approximately 30% [18].

The second point is the heterogeneity in renal structure and pathogenesis of type 2 diabetes. Due to the heterogeneity of type 2 diabetes, a minority of type 2 diabetic patients have typical histopathological patterns resembling those present in type 1 diabetes, due to the heterogeneity of type 2 diabetes. Only 30% of type 2 diabetic patients with microalbuminuria and 50% of patients with proteinuria demonstrate typical diabetic glomerulopathy [23]. Atypical patterns of renal injury account for 35% of those with microalbuminuria and proteinuria. These patients exhibiting mild or atypical diabetic glomerular lesions usually present severe tubulointerstitial lesions which include tubular atrophy, TBM thickening and reduplication, advanced glomerular arteriolar hyalinosis associated with atherosclerosis of large vessels, interstitial fibrosis, and global glomerular sclerosis. These tubulointerstitial lesions not only are related to hyperglycemia, but also reflect the contributions from various causes predated type 2 diabetes, such as ageing, atherosclerosis, and systemic hypertension [23].

## 5. Differential Diagnosis of DN with another Nodular Glomerulopathy

Nodular diabetic glomerulosclerosis has a variety of pathological features but still should be differentiated from another mesangial nodular sclerosing glomerulopathy, which usually has similar light microscopic manifestations. It is necessary and essential to include manifestations, immunofluorescence staining (IF), and EM into the distinction of lesions caused by immune complex or monoclonal protein [26].

Nodular lesions could be observed in various renal primary and secondary diseases, such as membranoproliferative glomerulonephritis, renal amyloidosis, type III collagen glomerulopathy, monoclonal immunoglobulin or light chain deposition disease, fibronectin nephropathy, and cryoglobulinemia glomerulosclerosis [6, 27–29].  Table 3 shows the differential diagnosis of several mesangial nodular sclerosing glomerulopathy based on clinical manifestations, periodic acid Schiff (PAS) stain, methenamine silver stain, IF, and EM.

## 6. DM Accompanied with Nondiabetic Renal Disease (NDRD)

DM patients generally do not receive renal biopsy, unless there is a need to make an exclusive or differential diagnosis due to the complicated clinical situation. Because most of the DM patients undergoing renal biopsy show compound clinical manifestations, it is easy for pathologists to find superimposed lesions in renal biopsy in addition to pure pathological changes of DN.

Normally, no immune complexes and obvious complements could be detected by IF and EM in patients with DN. If there is a variety of typical deposition in the glomeruli, such as granular/chunky patterns of immunoglobulin by IF or electron dense deposits by EM, it usually indicates a superimposed nondiabetic renal disease [6].

In China, IgA nephropathy was the most frequently biopsy finding seen in all NDRD patients, followed by membranous nephropathy, mesangial proliferative glomerulonephritis, hypertensive nephrosclerosis, renal damage, minimal-change disease, focal segmental glomerulosclerosis, and crescentic glomerulonephritis [30]. However, the disease spectrum of NDRD varies in different populations. For example, in the United States, unlike in China which has a high prevalence of IgA nephropathy, two large-scale retrospective studies found that focal segmental glomerulosclerosis, acute tubular necrosis, and IgA nephropathy were the most common lesions found in patients with NDRD. Hypertensive nephrosclerosis, minimal-change disease, and membranous nephropathies were also common NDRDs of diabetic patients in the United States [31, 32]. In this review, we focus on the prevalence of NDRD in China.  Table 4 summarizes recent studies on NDRD which include both NDRD alone and alongside concomitant DN in China.

As the DN and NDRD have different causes, their relationship, synergistic or independent, remains to be further studied. Given the fact that there is a wide clinical variation of DN patients combined with NDRD, the renal biopsy is an important method to improve the detection rate of NDRD. A clear diagnosis of the renal disease and proactive treatment meanwhile can stabilize or even reverse the renal function and improve the long-term prognosis of patients.

## 7. The Indications for Renal Biopsy

Although renal biopsy is the gold standard of DN diagnosis, the majority of diabetic patients with renal involvement are not biopsied. Some scholars believe that most diabetic glomerular changes are nonspecific in the early stage of diabetes, so there is no need to expand the indications of renal biopsy blindly. The bleeding risk of renal biopsy should be carefully considered in patients who have been suffering from hypertension, renal dysfunction, or anemia [33].

Moreover, microalbuminuria is clinically considered as a major index to judge the progression of DN. However, it is not as accurate as expected. For example, type 1 diabetes usually develops into DN within 10 to 15 years after diagnosis, while microalbuminuria may occur as early as 2 to 5 years after diagnosis. Some patients with type 2 diabetes may already have microalbuminuria at the time of diagnosis, but without DN. Thus, renal biopsy and morphological changes may offer important insights into the understanding of the complex course of diabetes and help to classify, diagnose, prognose, and manage the disease. Indications for renal biopsy in DN are as follows [30, 34]:

1. Proteinuria of nephrotic range with diabetes less than 5 years or normal kidney functions
2. An unexplained microscopic hematuria (especially acanthocytosis and cellular cast)
3. An unexplained rapidly worsening renal function in patients with a previously stable renal function
4. Application of angiotensin converting enzyme inhibitor (ACEI)/angiotensin receptor antagonist (ARB) for 2~3 months, while GFR decreased by more than 30%
5. Failure to exclude renal diseases in the absence of diabetic retinopathy, with or without systemic diseases

## 8. Conclusion

DN has a pathological diversity and affects all structural components of the kidney. Recognition of these lesions and their morphological characteristics in renal biopsy may aid in preventing, slowing down, or even reversing the processes of diabetic nephropathy. Tervaert classification of DN has a positive meaning in developing novel strategies for an early diagnosis and treatment of DN. However, the complexity and heterogeneity of type 2 diabetes, different from type 1 diabetes, along with tubular, interstitial, and vascular lesions should be taken into consideration in new classification methods in the future. Regardless of its limitations, the Tervaert classification represents a very meaningful step towards the establishment of a DN classification scheme with clinical utility. When the pathologist observes the pathological changes of DN, they still need to make a differential diagnosis with another nodular glomerulopathy and clarify whether it is a typical DN complicated with NDRD or not. Furthermore, the indications and risks of renal biopsy should be prudently taken into consideration.

## Figures and Tables

**Table 1:** Diabetic nephropathy is divided into four hierarchical glomerular lesions.

Class	Description and criteria
I	Mild or nonspecific changes on light microscopy and conformed GBM thickening proven by electron microscopy: GBM > 395 nm (female), GBM > 430 nm (male).
IIa	Mild mesangial expansion in >25% of the observed mesangium; area of mesangial proliferation < area of capillary cavity.
IIb	Severe mesangial expansion in >25% of the observed mesangium. Area of mesangial proliferation < area of capillary cavity.
III	At least one convincing nodular sclerosis (Kimmelstiel-Wilson lesion).
IV	Advanced diabetic glomerulosclerosis in >50% of glomeruli.

**Table 2:** Separate scoring system of interstitial and vascular lesions of DN.

Lesion	Criteria	Score
Tubulointerstitial lesions	No IFTA	0
	IFTA < 25%	1
	25% < IFTA < 50%	2
	IFTA > 50%	3

Interstitial inflammation	Absent	0
	Relate to IFTA	1
	In areas without IFTA	2

Arteriolar hyalinosis	Absent	0
	One hyaline arteriole	1
	More than one hyaline arteriole	2

Arteriosclerosis (most severely affected artery)	No intimal thickening is observed	0
	Intimal thickening is less than the thickness of the media	1
	Intimal thickening is more than the thickness of the media	2

*Note.* IFTA: tubulointerstitial fibrosis and tubular atrophy.

**Table 3:** Differential diagnosis of DN with another nodular glomerulopathy.

Disease	Clinical manifestations	Light microscopy	Immunofluorescence microscopy	Electron microscopy
Diabetic nephropathy	Long duration of diabetes	Mesangial nodular sclerosis; PAS (+); silver (+)	Linear deposition of immunoglobulin (Ig) G, with or without IgM and C3 in sclerotic nodules	Mesangial expansion; diffuse GBM thickening; nonspecific fibrillar deposition

Membranoproliferative glomerulonephritis	Chronic nephritis, nephrotic syndrome, hypertension, hypocomplementemia	Mesangial nodular sclerosis; mesangial insertion; double contouring; PAS (+); silver (+)	Granular deposition of multiple immunoglobulin deposition and complement components	Subendothelial (type I), intramembranous, often ribbon-like or nodular (type II), subepithelial (type III) electron-dense deposits

Renal amyloidosis	Chronic infection, systemic amyloidosis, lymphoproliferative disease, long-term dialysis, family inheritance	Mesangial nodular sclerosis; Congo red (+)	Light chain *λ* (+) in the mesangium, GBM, tubulointerstitium, and blood vessel wall	Amyloid fibrils (randomly oriented, nonbranching, 9−11 nm in diameter)

Monoclonal immunoglobulin/light chain deposition disease	Ageing, plasma cell dyscrasia, idiopathic	Mesangial nodular sclerosis; PAS (+); silver (−)	Monoclonal light chain *κ* (+) in the GBM, TBM, and vascular wall basement membranes	Granular, powdery deposits

Type III collagen glomerulopathy	Persistent proteinuria, nephrotic syndrome	Mesangial nodular sclerosis; PAS (weak +)	Collagen III (+)	Parallel collagen fibers (100 nm in diameter)

Fibronectin nephropathy	A rare autosomal dominant disease, nephrotic syndrome	Mesangial nodular sclerosis; PAS (+); Congo red (−)	Fibronectin (+)	Granular deposits with short fibers (10−14 nm in diameter)

Cryoglobulinemia glomerulosclerosis	Proteinuria, nephrotic syndrome, high serum cryoglobulins, lymphoproliferative disorders	Intracapillary proliferation and inflammatory cell infiltrates, intracapillary thrombi, nodular glomerulosclerosis; double contouring; PAS (+)	Monoclonal or polyclonal immunoglobulin (IgM, IgG and C3), rheumatoid factor	Organized electron-dense deposits (microtubular, 30 nm in diameter)

Idiopathic nodular glomerulosclerosis	Smoking, long-standing hypertension; normal glucose metabolism	Similar to those of nodular diabetic glomerulosclerosis,	IgG and albumin	No electron-dense or fibrillar deposits

**Table 4:** Literature review of NDRD in China.

Author	Year of publication	References	Number of patients	Pathological diagnosis			Statistical analysis of NDRD (alone and coexistent with DN)
				DN	NDRD	DN plus NDRD	
Mak et al.	1997	[35]	51	67	16	17	IgAN (59%), HTN (24%)

Wong et al.	2002	[36]	68	35	46	19	IgAN (29.5%), HTN (20.4%), MN (18.2%), MCD (9%)

Cao et al.	2007	[37]	120	85.8	—	14.2	IgAN (41.2%), MN (17.6%), HTN (11.7%), TIN (11.7%), RA (5.9%), FSGS (5.9%), micropolyarteritis (5.9%)

Zhou et al.	2008	[38]	110	54.5	—	45.5	IgAN (34%), MN (22%), MPGN (14%), HBV-associated GN (8%), MCD (4%), HTN (4%), MPGN (4%), CrGN (2%)

Lin et al.	2009	[39]	50	48	22	30	MN (19.23%), IgAN (11.54%), MCD (3.85%), HTN (3.85%), ATN (3.85%), CIN (7.69%)

Mou et al.	2010	[40]	69	47.8	52.2	—	FSGS (37.7%), IgAN (15.9%), MCD (15.9%), MN (8.7%)

Bi et al.	2011	[41]	220	54.5	—	45.5	IgAN (34%), MN (22%), MPGN (14%), HBV-associated GN (8%), MCD (4%), HTN (4%), MPGN (4%), CrGN (2%)

Zhang et al.	2011	[42]	130	73.9	26.1	—	IgAN (16.9%), MN (6.15%)

Zhuo et al.	2013	[43]	216	6.5	82.9	10.7	17–35 years (group I): IgAN (29%), MN (11.8%), FSGS (8.8%), MPGN (8.8%), APGN (5.9%), CrGN (5.9%); 36–59 years (group II): IgAN (34.7%), MN (15%), FSGS (1.4%), MPGN (6.1%), APGN (0.7%), CrGN (1.4%); 60 years (group III): IgAN (2.9%), FSGS (2.9%), MN (25.7%), CrGN (8.6%), MPGN (11.4%), RA (5.7%)

Peng and Wang	2013	[44]	61	52.5	—	47.5	IgAN (31%), MN (17.2%), MPGN (13.8%), HTN (13.8%), FSGS (10.3%), MCD (6.9%), APGN (3.5%)

Wang	2015	[45]	56	57.1		42.9	IgAN (33.3%), MN (25.0%), MPGN (20.8%), HTN (8.3%), FSGS (4.2%), MCD (4.2%)

*Note.* IgAN, primary IgA nephropathy; HTN, hypertensive nephrosclerosis; MN, membranous nephropathy; MCD, minimal change disease; TIN, tubulointerstitial nephritis; RA, renal amyloidosis; MPGN, mesangial proliferative glomerulonephritis; CrGN, crescentic glomerulonephritis; ATN, acute tubular necrosis; CIN, chronic interstitial nephritis; FSGS, focal segmental glomerular sclerosis.

## References

[1] Zou W., Wang H. (2009). *Pathology of Renal Biopsy*.

[2] KDOQI (2007). KDOQI clinical practice guidelines and clinical practice recommendations for diabetes and chronic kidney disease. *American Journal of Kidney Diseases*.

[3] Radcliffe N. J., Seah J.-M., Clarke M., Macisaac R. J., Jerums G., Ekinci E. I. (2016). Clinical predictive factors in diabetic kidney disease progression. *Journal of Diabetes Investigation*.

[4] An Y., Liu Z. (2013). Relationship between pathological changes and prognosis in diabetic nephropathy. *Chinese Journal of Nephrology, Dialysis & Transplantation*.

[5] Maezawa Y., Takemoto M., Yokote K. (2015). Cell biology of diabetic nephropathy: roles of endothelial cells, tubulointerstitial cells and podocytes. *Journal of Diabetes Investigation*.

[6] Najafian B., Alpers C. E., Fogo A. B. (2011). Pathology of human diabetic nephropathy. *Diabetes and the Kidney*.

[7] Pourghasem M., Shafi H., Babazadeh Z. (2015). Histological changes of kidney in diabetic nephropathy. *Caspian Journal of Internal Medicine*.

[8] Caramori M. L., Kim Y., Huang C. (2002). Cellular basis of diabetic nephropathy: 1. Study design and renal structural-functional relationships in patients with long-standing type 1 diabetes. *Diabetes*.

[9] Nosadini R., Velussi M., Brocco E. (2000). Course of renal function in type 2 diabetic patients with abnormalities of albumin excretion rate. *Diabetes*.

[10] Tervaert T. W. C., Mooyaart A. L., Amann K. (2010). Pathologic classification of diabetic nephropathy. *Journal of the American Society of Nephrology*.

[11] Hong D., Zheng T., Jia-qing S., Jian W., Zhi-hong L., Lei-shi L. (2007). Nodular glomerular lesion: a later stage of diabetic nephropathy?. *Diabetes Research and Clinical Practice*.

[12] Fioretto P., Mauer M. (2007). Histopathology of diabetic nephropathy. *Seminars in Nephrology*.

[13] Drummond K. N., Kramer M. S., Suissa S. (2003). Effects of duration and age at onset of type 1 diabetes on preclinical manifestations of nephropathy. *Diabetes*.

[14] Gilbert R. E., Cooper M. E. (1999). The tubulointerstitium in progressive diabetic kidney disease: more than an aftermath of glomerular injury?. *Kidney International*.

[15] Min W., Yamanaka N. (1993). Three-dimensional analysis of increased vasculature around the glomerular vascular pole in diabetic nephropathy. *Virchows Archiv A Pathological Anatomy and Histopathology*.

[16] White K. E., Bilous R. W. (2004). Structural alterations to the podocyte are related to proteinuria in type 2 diabetic patients. *Nephrology Dialysis Transplantation*.

[17] Najafian B., Crosson J. T., Kim Y., Mauer M. (2006). Glomerulotubular junction abnormalities are associated with proteinuria in type 1 diabetes. *Journal of the American Society of Nephrology*.

[18] Parving H.-H., Gall M.-A., Skøtt P. (1992). Prevalence and causes of albuminuria in non-insulin-dependent diabetic patients. *Kidney International*.

[19] Fioretto P., Caramori M. L., Mauer M. (2008). The kidney in diabetes: dynamic pathways of injury and repair. The Camillo Golgi Lecture 2007. *Diabetologia*.

[20] Zhu X., Liu F., Peng Y. (2012). New pathologic classification of diabetic nephropathy (retrospective study of 37 cases). *Journal of Central South University (Medical Sciences)*.

[21] Oh S. W., Kim S., Na K. Y. (2012). Clinical implications of pathologic diagnosis and classification for diabetic nephropathy. *Diabetes Research and Clinical Practice*.

[22] An Y., Xu F., Le W. (2015). Renal histologic changes and the outcome in patients with diabetic nephropathy. *Nephrology Dialysis Transplantation*.

[23] Fioretto P., Mauer M., Brocco E. (1996). Patterns of renal injury in NIDDM patients with microalbuminuria. *Diabetologia*.

[24] Ekinci E. I., Jerums G., Skene A. (2013). Renal structure in normoalbuminuric and albuminuric patients with type 2 diabetes and impaired renal function. *Diabetes Care*.

[25] Fioretto P., Mauer M. (2010). Diabetic nephropathy: diabetic nephropathy-challenges in pathologic classification. *Nature Reviews Nephrology*.

[26] Markowitz G. S., Lin J., Valeri A. M., Avila C., Nasr S. H., D'Agati V. D. (2002). Idiopathic nodular glomerulosclerosis is a distinct clinicopathologic entity linked to hypertension and smoking. *Human Pathology*.

[27] Guo M. (2007). *Pathology of Renal Biopsy*.

[28] Kumar V., Abbas A. K., Aster J. C. (2012). *Robbins Basic Pathology*.

[29] Alsaad K. O., Herzenberg A. M. (2007). Distinguishing diabetic nephropathy from other causes of glomerulosclerosis: an update. *Journal of Clinical Pathology*.

[30] Fiorentino M., Bolignano D., Tesar V. (2016). Renal biopsy in 2015—from epidemiology to evidence-based indications. *American Journal of Nephrology*.

[31] Sharma S. G., Bomback A. S., Radhakrishnan J. (2013). The modern spectrum of renal biopsy findings in patients with diabetes. *Clinical Journal of the American Society of Nephrology*.

[32] Pham T. T., Sim J. J., Kujubu D. A., Liu I.-L. A., Kumar V. A. (2007). Prevalence of nondiabetic renal disease in diabetic patients. *American Journal of Nephrology*.

[33] Espinel E., Agraz I., Ibernon M., Ramos N., Fort J., Serón D. (2015). Renal biopsy in type 2 diabetic patients. *Journal of Clinical Medicine*.

[34] Chen J. (2011). Early diagnosis of diabetic nephropathy and indications of renal biopsy. *Nephrology Dialysis Transplantation*.

[35] Mak S. K., Gwi E., Chan K. W. (1997). Clinical predictors of non-diabetic renal disease in patients with non-insulin dependent diabetes mellitus. *Nephrology Dialysis Transplantation*.

[36] Wong T. Y. H., Choi P. C. L., Chun C. S. (2002). Renal outcome in type 2 diabetic patients with or without coexisting nondiabetic nephropathies. *Diabetes Care*.

[37] Cao S., Fang M., Sun Y., Fang Y., Liu Z. (2007). Clinical and pathological analysis of diabetic nephropathy combined with non-diabetic nephropathy. *Clinical Focus*.

[38] Zhou J., Chen X., Xie Y., Li J., Yamanaka N., Tong X. (2008). A differential diagnostic model of diabetic nephropathy and non-diabetic renal diseases. *Nephrology Dialysis Transplantation*.

[39] Lin Y.-L., Peng S.-J., Ferng S.-H., Tzen C.-Y., Yang C.-S. (2009). Clinical indicators which necessitate renal biopsy in type 2 diabetes mellitus patients with renal disease. *International Journal of Clinical Practice*.

[40] Mou S., Wang Q., Liu J. (2010). Prevalence of non-diabetic renal disease in patients with type 2 diabetes. *Diabetes Research and Clinical Practice*.

[41] Bi H., Chen N., Ling G., Yuan S., Huang G., Liu R. (2011). Nondiabetic renal disease in type 2 diabetic patients: a review of our experience in 220 cases. *Renal Failure*.

[42] Zhang P.-P., Ge Y.-C., Li S.-J., Xie H.-L., Li L.-S., Liu Z.-H. (2011). Renal biopsy in type 2 diabetes: timing of complications and evaluating of safety in Chinese patients. *Nephrology*.

[43] Zhuo L., Ren W., Li W., Zou G., Lu J. (2013). Evaluation of renal biopsies in type 2 diabetic patients with kidney disease: a clinicopathological study of 216 cases. *International Urology and Nephrology*.

[44] Peng L., Wang Y. (2013). Pathological analysis of diabetic nephropathy and non-diabetic renal disease in patients with type 2 diabetes mellitus. *Chinese and Foreign Medical Research*.

[45] Wang X. (2015). Pathological and clinical characteristics of diabetic nephropathy and diabetic nephropathy combined with non-diabetic nephropathy. *Journal of Clinical Research*.

